# Acceptabilité du dépistage néonatal de la drépanocytose au cours de la pandémie au COVID-19 à Kisangani, en République Démocratique du Congo

**DOI:** 10.11604/pamj.2020.37.299.26654

**Published:** 2020-12-02

**Authors:** Emmanuel Tebandite Kasai, Jean Pierre Alworong´a Opara, Salomon Batina Agasa, Béatrice Gulbis, Naura Apio Uvoya, Jean Didier Bosenge Nguma, Philippe Kasongo Maloba, Philippe Hubert, Anne-Marie Etienne, Roland Marini Djang´eing´a

**Affiliations:** 1 Département de Pédiatrie, Faculté de Médecine et de Pharmacie, Université de Kisangani, Kisangani, République Démocratique du Congo,; 2 Département de Médecine Interne, Faculté de Médecine et de Pharmacie, Université de Kisangani, Kisangani, République Démocratique du Congo,; 3 Département de Chimie Clinique, Hôpital Erasme, Université Libre de Bruxelles, Bruxelles, Belgique,; 4 Département de Pédiatrie, Faculté de Médecine, Université de Bunia, Bunia, République Démocratique du Congo,; 5 Département de Gynécologie-Obstétrique, Faculté de Médecine et de Pharmacie, Université de Kisangani, Kisangani, République Démocratique du Congo,; 6 Département de Psychologie, Faculté de Psychologie et des Sciences de l´Education, Université de Lubumbashi, Lubumbashi, République Démocratique du Congo,; 7 Laboratoire de Chimie Analytique Pharmaceutique, Center for Interdisciplinary Research on Medicines (CIRM), Faculté de Médecine, Université de Liège, Liège, Belgique,; 8 Unité de Psychologie de la Santé, Faculté de Psychologie, Logopédie et Sciences de l´Education, Université de Liège, Liège, Belgique,; 9 Département de Pharmacie, Faculté de Médicine et de Pharmacie, Université de Kisangani, Kisangani, République Démocratique du Congo

**Keywords:** Drépanocytose, dépistage néonatal, hemotypeSC^TM^, acceptabilité, COVID-19, Kisangani, RDC, Sickle cell disease, neonatal screening, HemotypeSC^TM^, acceptability, COVID-19, Kisangani, DRC

## Abstract

**Introduction:**

l´implémentation du dépistage néonatal de la drépanocytose pendant la pandémie de coronavirus (COVID-19) représente un défi majeur en République Démocratique du Congo (RDC). La présente étude vise à déterminer si des facteurs socio-économiques sont associés à l´acceptabilité du dépistage néonatal de la drépanocytose pendant la COVID-19 à Kisangani, en RDC.

**Méthodes:**

étude observationnelle conduite dans les maternités de Kisangani du 21 mars au 30 juin 2020 chez les mères sensibilisées au dépistage néonatal de la drépanocytose de leurs nouveau-nés à l´hemotypeSC^TM^ (HT401RUO-USA). Les données recueillies étaient la parité, le niveau d´étude, l´âge, le niveau socio-économique, la profession, la notion de sensibilisation et le motif du refus du dépistage.

**Résultats:**

sur 55,5% (273/492) des mères sensibilisées, 107 (39,19 %) ont accepté et 166 (60,80 %) ont refusé le dépistage néonatal de la drépanocytose chez leur nouveau-né. Les motifs du refus étaient l´absence d´information (67,5%; IC 95% [59,8-74,5]), le manque d´argent dû au confinement (66,3%; IC 95% [58,5-73,4]), la prise de sang pour tentative du vaccin anti-COVID-19 (63,2%; IC 95% = [55,4-70,6]). Les facteurs associés à l´acceptabilité du dépistage étaient l´âge > 35 ans (p = 0,0009; ORa = 3,04; IC 95% = 1,57-5,87) et le bas niveau socio-économique (p = 0,0016; ORa = 2,29; IC à 95% = 1,37-3,85).

**Conclusion:**

l´acceptabilité du dépistage néonatal de la drépanocytose pendant la COVID-19 reste faible à Kisangani. Le gouvernement devrait identifier les canaux de communication efficaces afin de promouvoir les initiatives dans le secteur de la Santé.

## Abstract (en)

**Introduction:**

the implementation of neonatal screening to identify infants with sickle cell disease during the COVID-19 pandemic is a major challenge in the Democratic Republic of the Congo (DRC). The purpose of this study is to determine whether socio-economic factors are associated with acceptability of newborn screening to identify infants with sickle cell disease during the COVID-19 pandemic in Kisangani, DRC.

**Methods:**

we conducted an observational study of mothers sensitized to neonatal screening to detect sickle cell disease in their newborns with hemotypeSC^TM^ (HT401RUO-USA). The study was carried out at the maternity wards in Kisangani from March 21^st^ to June 30^th^ 2020. Collected data were parity, educational level, age, socio-economic level, occupation, awareness and the reason for the denial of screening.

**Results:**

out of 55.5% (273/492) of sensitized mothers, 107 (39.19%) accepted and 166 (60.80%) refused neonatal screening to detect sickle cell disease in their newborn. The reasons for refusal were lack of information (67.5%; 95% CI [59.8-74.5]), lack of money due to confinement (66.3%; 95% CI [58.5-73.4]), blood test to develop a vaccine for protection against COVID-19 (63.2%; 95% CI = [55.4-70.6]). Factors associated with the acceptability of screening were age > 35 years (p = 0.0009; ORa = 3.04; 95% CI = 1.57-5.87) and low socio-economic level (p = 0.0016; ORa = 2.29; 95% CI = 1.37-3.85).

**Conclusion:**

the acceptability of neonatal screening to detect sickle cell disease during COVID-19 is low in Kisangani. The government should identify effective communication channels to promote health care initiatives.

## Introduction

La drépanocytose constitue un problème crucial de Santé Publique dans le monde [1]. Par sa chronicité et la sévérité des crises qu´elle engendre, son suivi médical et social s´avère nécessaire [2]. Elle représente un lourd fardeau pour l´Afrique subsaharienne [1] où naissent chaque année 75% des nouveau-nés qui en sont atteints [3]. La République Démocratique du Congo (RDC) en est largement touchée avec une prévalence qui la classe 2^e^ pays en Afrique après le Nigéria et 3^e^ au monde après l´Inde et le Nigeria [4]. Des études rapportent environ 1,5% des homozygotes et 20 à 40% des hétérozygotes dans la population Congolaise [5]. Trente mille à quarante mille nouveau-nés SS y naissent chaque année [6] ce qui en porte la prévalence néonatale à 1,4% [5]. Le programme de dépistage néonatal systématique devrait y être implanté pour identifier précocement les enfants atteints et les prendre en charge correctement [6]. A Kisangani, la prévalence néonatale du trait drépanocytaire varie entre 18,3% et 23,3% [7, 8] tandis que celle de la maladie avoisine le 1% [7]. Cependant, Agasa *et al*. signalent une faible sensibilisation, ce qui explique l´orientation des enfants atteints vers d´autres structures (églises, guérisseurs) que celles de santé [9]. Il n´y a pas longtemps, les tests rapides de dépistage (TDR) étaient quasi inexistants à Kisangani [8, 10]. Ceci avait comme conséquence une prise en charge inadéquate ou tardive de la maladie [8, 9].

Deux études ont rapporté la validation analytique de deux tests rapides de dépistage de la drépanocytose dont l´HemotypeSC^TM^ chez les nouveau-nés et le Sickle Scan® chez les femmes enceintes [8, 10]. Les résultats de ces études ont démontré une sensibilité et une spécificité variant respectivement de 98,39% à 100% et de 96,3 à 99,43% [8]. Comparé à 15$, frais payés par les gestantes pour les examens prénuptiaux, le coût unitaire de ce test s´élève à 0,5$, ce qui est financièrement accessible dans un pays aux ressources limitées. En vue d´améliorer la prise en charge de cette maladie à Kisangani [9, 11], nous avons initié le dépistage néonatal systématique à l´HemotypeSC^TM^ et la sensibilisation des leaders communautaires. Ce dépistage était lancé après deux séries de formation des prestataires de soins couplées à une séance de sensibilisation des leaders communautaires. Ces activités ont été appuyées par le projet de recherche pour le developpement-2018 (PRD-2018) dénommé «DREPAKIS: contribution à la prise en charge de la drépanocytose dans la ville de Kisangani» qui a pour but d´appuyer les activités de recherche visant à améliorer la prise en charge des drépanocytaires dans la ville. Pendant la mise en œuvre du dépistage, la pandémie de COVID-19 a débuté et, à cause de la rapidité de sa propagation et de sa létalité, a constitué une réelle menace pour la Santé Publique [12, 13]. Elle est considérée comme la fin du monde par une frange de la population [14, 15]. Des mesures ont été prises pour ralentir cette propagation parmi lesquelles la mise en quarantaine, l´interdiction des rassemblements et la fermeture des lieux publics (églises, écoles, restaurants, buvettes, etc.) [15]. Cependant, ces lieux constituent souvent les canaux efficaces de communication de masse dans la promotion des activités telles que la vaccination et les tests de dépistage étant donné la difficulté d´accès aux autres sources d´information sûres (radio, télévision, journaux) à Kisangani [12, 16].

L´état d´urgence sanitaire, décrété le 10 mars 2020 par l´Organisation Mondiale de la Santé (OMS) et ensuite relayé en RDC par ses autorités le 19 mars 2020, a provoqué une panique générale [14, 15, 17]. De fausses rumeurs ont commencé à circuler dans les réseaux sociaux stipulant la retenue de la RDC pour l´expérimentation d´un éventuel vaccin anti-COVID-19 [18]. Ces fausses rumeurs étaient en contraste avec l´attente de la population Congolaise d´être informée par les canaux officiels tels que la télévision, concernant les malades affectés par le COVID-19 à l´instar d´autres pays comme les Etats-Unis, la France, l´Italie, la Belgique, etc. Il va en découler le rejet, par la population de tout acte médical à caractère préventif (dépistage, vaccination, etc.) sous prétexte de se mettre à l´abri de toute tentative d´expérimentation du vaccin contre le COVID-19. Cette attitude a coïncidé avec notre proposition aux mères de réaliser le dépistage néonatal de la drépanocytose, qui comme attendu, a été refusée à cause des facteurs liés au COVID-19 d´une part, et à l´expérience relevée avec le vaccin contre Ebola au Nord-Est de la RDC d´autre part. Lors de cette dernière situation, la cause du refus par la population était la fausse rumeur faisant état d´un vaccin ayant la capacité de réduire la reproduction des congolais et d´affecter négativement les facultés mentales [19]. Ainsi, nous voudrions savoir si la faible sensibilisation par manque d´accès aux sources d´information sûres liées au confinement et à la conjoncture économique consécutive à la COVID-19, ne peuvent-elles pas affecter l´acceptabilité du dépistage néonatal de la drépanocytose par les mères. L´objectif de cette étude est de déterminer si des facteurs socio-économiques sont associés à l´acceptabilité du dépistage néonatal de la drépanocytose pendant la pandémie de COVID-19 à Kisangani.

## Méthodes

**Cadre de l´étude:** cette étude s´est déroulée dans la ville de Kisangani, chef-lieu de Province de la Tshopo, située au Nord-Est de la RDC. Les données étaient recueillies dans les Zones de Santé (ZS) de Kabondo et de Makiso-Kisangani qui présentent une configuration urbano-rurale. Ces deux ZS couvrent une population totale de 558.786 habitants en raison de 22.351 nouveau-nés attendus par an, soit 1.862 nouveau-nés par mois. Les structures sanitaires retenues pour cette étude sont celles qui avaient également servi de cadre d´une étude pilote conduite en mars 2020 pour valider l´HemotypeSC^TM^ comme test de dépistage rapide de la drépanocytose chez les nouveau-nés [8]. Il s´agit des Centres de Santé (CS) Imani, Jamaa, Saint Camille, du Centre Hospitalier (CH) Nouveau Village de Pédiatrie et des Hôpitaux Généraux de Référence (HGR) de Kabondo et de Makiso-Kisangani. Cette étude a été autorisée par le comité d´éthique de l´Université de Kisangani (N°UNIKIS/CER/005/2018).

**Cohorte de l´étude:** il s´agit d´une étude observationnelle conduite du 21 mars au 30 juin 2020 à Kisangani, incluant des femmes en post-partum qui ont été présentes dans l´une des structures sanitaires reprises ci-haut, et qui par ailleurs avaient accepté le dépistage de la drépanocytose chez leur nouveau-né moyennant le consentement à l´issue de la sensibilisation. Nous avons également inclus dans la cohorte les femmes qui, au départ, avaient refusé de participer, mais par la suite ont marqué leur accord en donnant les raisons au cours des dialogues individuels.

**Déroulement de l´enquête:** une visite de sensibilisation à la vaccination et au dépistage de la drépanocytose chez les nouveau-nés a été réalisée dans toutes les maternités de l´étude, 72 heures post-accouchement. L´enquêteur animait, pendant 15 à 20 minutes et en petits groupes de 4 à 5 mères, une séance de sensibilisation en Lingala ou en Swahili, deux langues couramment parlées à Kisangani. Le message consistait à définir la drépanocytose, à différencier un porteur du trait d´un malade, à donner l´ampleur de la maladie y compris ses conséquences, à expliquer le pourquoi du dépistage néonatal systématique ainsi que ses avantages et à déterminer l´âge moyen de la survenue des signes révélateurs de la maladie. Les mères étaient informées que le dépistage du nouveau-né était conditionné à un coût équivalent à 0,5$. Ce montant a été fixé conformément aux réalités socio-économiques locales et de commun accord avec les leaders communautaires au cours d´une séance de sensibilisation organisée à leur intention. Un consentement éclairé verbal et écrit était demandé aux enquêtées.

**Procédure du dépistage:** le dépistage néonatal a été réalisé au moyen d´un test rapide du type HemotypeSC^TM^ (HT401RUO-USA). Un prélèvement de 1,5 µL du sang total au talon gauche du nouveau-né a été effectué pour la réalisation du test. Les différentes étapes de réalisation du test ainsi que le principe de lecture du résultat ont été décrits par Kasai *et al*. [8]. Le résultat était immédiatement communiqué à la mère par l´enquêteur après avoir clarifié les notions qui n´étaient pas bien comprises pendant la leçon en groupe. Il notait le résultat au crayon sur le canevas d´entretien ainsi que dans le registre préposé au bureau de l´infirmier responsable de la structure où il recevait individuellement les mères.

**Recueil des données:** les données suivantes des enquêtées ont été recueillies à savoir la parité, le niveau d´étude, l´âge, le niveau socio-économique, l´état matrimonial, la profession, la notion de sensibilisation antérieure et la raison du refus du dépistage. Pour cela, nous nous sommes servi d´un canevas d´entretien reprenant les questions fermées et établi en référence à la littérature sur les obstacles aux services médicaux pour les patients atteints de la drépanocytose [20]. Par ailleurs, l´étude était conduite suivant les recommandations de *STROBE GUIDELINES* sur le reportage d´une étude observationnelle [21]. Pour permettre d´avoir le contrôle sur le processus, l´enquêteur sensibilisait lui-même les mères, faisait le dépistage et leur communiquait le résultat.

**Analyses statistiques:** elles ont été réalisées au moyen du logiciel Epi info® 7.2.2.6 (CDC, 2018). Les variables catégorielles ont été présentées en effectifs et en pourcentages. La comparaison des proportions a été effectuée à l´aide du test Chi-2 de Pearson et de Test exact de Fisher. La force d´association statistique entre l´acceptabilité du dépistage et les différentes caractéristiques maternelles était évaluée par le calcul des odds ratios (OR) bruts (en analyses bivariées) et des odds ratios ajustés (en analyses multivariées) ainsi que leurs intervalles de confiance (IC) à 95%. Nous avons fixé le seuil de signification à la P-value < 0,05.

**Définition des variables:** pour la parité, nous avons défini comme pauci-pare (parité 1 à 2), multipare (parité 3 à 4) et grande multipare (parité >4) [22]. Le niveau socio-économique était classé comme bas (absence de salaire ou de source de revenus clairement définie ou ne disposant ni de téléphone ni de trousse d´accouchement), moyen (avec ou sans salaire mais disposant d´un téléphone fonctionnel et d´une trousse d´accouchement) et élevé (salaire disponible avec avantages liés à la fonction, disposant d´un téléphone fonctionnel et d´une trousse d´accouchement) [23]. Le statut marital était classé comme marié (une femme qui vit en union) et célibataire (celle qui vit seule) [22]. Le niveau d´études était défini comme primaire (études primaires avec ou sans certificat), secondaire (études secondaires ou diplôme de fin d´études secondaires) et supérieur (études universitaires avec ou sans diplôme) [16, 23]. Pour la profession on classait fonctionnaire (travail rémunéré) et ménagère (travail non rémunéré) [16].

## Résultats

**Caractéristiques de la cohorte étudiée:** sur les 492 mères qui ont accouché pendant la période de l´étude, 273 (soit 55,5%) ont été sensibilisées pour la réalisation du dépistage de leurs nouveau-nés. Parmi celles-ci, un premier groupe de 107 femmes (soit 39,19 %) a accepté et un second groupe de 166 femmes (soit 60,80%) a refusé le dépistage néonatal de la drépanocytose chez leur enfant. La comparaison de différentes caractéristiques sociodémographiques des deux groupes sont présentées dans le Tableau 1.

**Tableau 1:** facteurs associés à l'acceptabilité du dépistage de la drépanocytose chez les nouveau-nés

Variables		Consentement au dépistage					
		Oui(n=107) N (%)	Non(n=166) N (%)	*P-value	Orb[IC à 95%]	**P-Value	ORa[IC à 95%]
Age	<18ans	7(0.93%)	11(6.62%)	0,9781	0,98[0,37-2,62]		
	[18et 35] ans	71(66.35%)	136(81.92%)	référence	1		
	>35ans	29(27.10%)	19(11.44%)	0,0009	2,87[1,51-5,45]	0,0009	3,04[1,57-5,87]
Parité	1à 2	60(56.07%)	81(48.79%)	0,2399	1,33[0,82-2,18]		
	3à 4	25(23.36%)	57(34.33%)	référence	1		
	>4	22(20.56%)	28(16.86%)	0,4411	1,27[0,68-2,37]		
NSE	Bas	54(50.46%)	52(31.32%)	0,0015	2,23[1,35-3,68]	0,0016	2,29[1,37-3,85]
	Moyen	34(37.77%)	91(54.81%)	référence	1		
	Elevé	19(17.75%)	23(13.85%)	0,3830	1,34[0,69-2,60]		
Instruction	Primaire	15(14.01%)	25(15.06%)	0,8122	0,91[0,46-1,83]		
	Secondaire	63(58.87%)	90(54.21%)	référence	1		
	Supérieur	29(27.10%)	51(30.72%)	0,5211	0,83[0,48-1,43]		
Profession	Ménagère	94(87.85%)	115(69.27%)	0,0004	3,20[1,64-6,24]	0,0658	0,57[0,32-1,32]
	Fonctionnaire	13(12.14%)	51(30.72%)	référence	1		
Etatcivil	célibataire	21(61,76)	13(38,24)	0,4560	1,04[0,50-2,24]		
	marié	145(60,67)	94(39,33)	Référence			

* P-value calculée en utilisant le chi-carré de Pearson ** P-value calculée en utilisant la régression statistique ORb: Odd Ratio brut / ORa: Odd Ratio ajustée

**Motifs d´un refus du dépistage néonatal de la drépanocytose:** parmi les 166 femmes qui avaient refusé de consentir au dépistage de la drépanocytose chez leurs nouveau-nés, 67,5% (n = 112; IC 95%: [59,8-74,5]) disent que l´information concernant le dépistage néonatal systématique n´a pas été annoncée par l´autorité du gouvernement, 66,3% (n = 110; IC 95%: [58,5-73,4]) déclarent n´avoir pas d´argent pour payer le test à cause de la situation économique précaire liée au confinement, 63,2% (n = 105; IC 95%: [55,4-70,6]) évoquent la prise du sang pour une tentative du vaccin anti-COVID-19 et 22,9% (n = 38; IC 95%: [17,74-30,04]) rapportent que les mesures préventives (vaccin, MILDA) étant gratuites en RDC par conséquent le test de dépistage de la drépanocytose devait également l´être. Le résumé des réponses des mères concernant les motifs du refus du dépistage est repris dans le Tableau 2.

**Tableau 2:** raisons évoquées par les mères pour motiver le refus de consentir au dépistage systématique de la drépanocytose chez leurs nouveau-nés

Raisons de refus du dépistage	N (166)	%	IC 95%
Manque d'argent à cause du confinement	110	66,27	[58,53-73,41]
Prise de sang pour tentative de vaccin anti-COVID-19/risque d'infecter l'enfant au COVID-19	105	63,25	[55,43-70,59]
Pas nécessaire car nouveau-né non malade	30	18,07	[12,54-24,78]
Pas de sensibilisation préalable par l'autorité	112	67,46	[59,78-74,53]
Peur d'un divorce	49	29,52	[22,70-37,08]
Activités préventives vraies sont non payables	38	22,89	[17,74-30,04]

**Acceptabilité du dépistage néonatal de la drépanocytose:** en analyse bivariée les facteurs associés à l´acceptabilité du dépistage de la drépanocytose chez les nouveau-nés étaient l´âge maternel supérieur à 34 ans, le bas niveau socio-économique, et la profession ménagère. Cependant, les résultats de la régression ont indiqué que seuls l´âge et le niveau socio-économique de la mère étaient associés à l´acceptabilité du dépistage de la drépanocytose chez le nouveau-né. Ainsi, l´acceptabilité était plus élevée chez les mères âgées de plus de 34 ans (P = 0,0009; ORa = 3,04; IC à 95%: 1,57-5,87) que chez les plus jeunes; ainsi que les mères avec un bas niveau socio-économique (P = 0,0016; ORa = 2,29; IC à 95%: 1,37-3,85).

## Discussion

Cette étude vise à déterminer les facteurs socio-économiques associés à l´acceptabilité du dépistage néonatal pendant la pandémie de COVID-19 à Kisangani. Tenant compte des résultats obtenus, il se dégage qu´un peu plus d´une mère sur deux (55,5%) a été sensibilisée. Ce chiffre faible peut s´expliquer par l´absence des canaux efficaces de communication liée à la pandémie de COVID-19 [12, 16]. En effet, le confinement avec fermeture des lieux publics et interdiction de regroupement, comme mesure préventive, ne permettait pas de sensibiliser les mères déjà psychologiquement stressées [24]. En plus, bien des mères, qui accouchaient, quittaient la maternité dans les 24 heures avant notre passage. Par ailleurs, la décision de sensibiliser était conditionnée par l´attitude générale des accouchées à notre arrivée [25]. Ces trois raisons n´ont pas permis de sensibiliser toutes les mères. Nous pensions qu´une sensibilisation ciblée aurait permis un taux plus important des mères sensibilisées par le fait d´informer les parents sur les conséquences de la drépanocytose tant pour l´enfant atteint que pour la famille voire la communauté tout en insistant sur les avantages de la prise en charge précoce qui n´est pas possible sans un dépistage très tôt dans la vie de l´enfant [26, 27].

Le taux d´acceptabilité du dépistage de 39,19% nous a paru assez faible comparativement aux données d´une récente étude pilote conduite à Kisangani qui a confirmé la favorabilité des mères au dépistage néonatal [8]. Dans une étude de sondage d´opinion réalisée au Nigéria, Teguete *et al*. avaient rapporté que 80% des mères acceptaient le dépistage néonatal de la drépanocytose chez leurs enfants [28]. Ce résultat supérieur au nôtre peut être expliqué entre autre par la gratuité du dépistage dans leur série [28], alors que nous avons mis en avant le caractère payant du test du dépistage. La conditionnalité du dépistage avait pour but de pérenniser cette activité. Par ailleurs, les conséquences économiques et financières décriées à travers le monde [29] n´ont pas épargné Kisangani et ont entretenu cet état des choses. Plusieurs facteurs peuvent expliquer le refus d´un programme de dépistage de masse d´une maladie chronique, dont la drépanocytose. Certains ont été évoqués par les mères mais d´autres comme le stress psychologique [17] et la peur de connaitre le statut de son enfant ou de soi-même n´ont pas été cités, pourtant ce sont des facteurs souvent avancés pour le refus d´accepter les activités du dépistage [30, 31].

Parmi les principales raisons évoquées par la majorité de nos enquêtées comme motif du refus de consentir au test de dépistage, on peut citer le manque d´argent pour payer le test à cause du confinement (66,3%), la crainte d´une prise du sang pour tentative de vaccin anti-COVID-19 ou par peur d´infecter le nouveau-né au COVID-19 (63,2%) et l´absence de sensibilisation préalable (67,46%). Le manque d´argent évoqué par les mères est consécutif à la crise économique et financière générée par la pandémie de COVID-19 dans le cadre du respect des mesures barrières [29]. Concernant la peur du vaccin et/ou le risque d´infecter les nouveau-nés, nous pouvons l´attribuer aux fausses rumeurs qui circulaient essentiellement sur les réseaux sociaux [15, 17]. Ces rumeurs faisaient croire à la population que la RDC était retenue parmi les pays où l´on devait expérimenter le vaccin anti-COVID-19 [18]. Soixante-sept pourcent des mères ont évoqué l´absence de sensibilisation préalable par l´autorité. Le manque de sensibilisation est un véritable obstacle à la réussite des campagnes de dépistage de beaucoup de maladies chroniques dont la drépanocytose [32].

Dans une résolution de l´assemblée générale des Nations Unies en 2008, il était fait état de préjugés néfastes à l´égard de la drépanocytose et de l´importance de sensibiliser le public [25]. Une résolution de l´UNESCO précise qu´un manque d´information est source des croyances surnaturelles sur la maladie, que la drépanocytose a un important retentissement social sur les familles et qu´elle constitue un facteur de division du tissu familial et d´exclusion de la société [33]. D´autres auteurs rapportent également que la compréhension de la maladie, de ses causes et de son mode de transmission demeure souvent teintée par les conceptions préexistantes véhiculées au sein de certaines communautés [20]. Pour ce qui est de la ville de Kisangani, la fermeture des lieux publics dont les églises et le non tenue des activités de consultation prénatale ou préscolaire en rapport avec la COVID-19 ont fait obstacle à la sensibilisation à tous les niveaux [12, 15, 16]. A ce sujet, il ressort de l´enquête démographique et de santé (EDS) 2013-2014 [16] qu´il n´est pas nécessaire que le ménage dispose de la radio, de la télévision ou achète un journal pour avoir accès aux informations car de nombreuses personnes peuvent écouter la radio ou regarder la télévision chez des amis ou des voisins. Le confinement avec le slogan *«restez chez vous et évitez les sorties inutiles»* a fait obstruction à ces genres de visite. Même alors si cela était possible, en RDC, il est prouvé que les proportions des femmes et des hommes habituellement exposés aux 3 médias sont très faibles de l´ordre de 2% et 6% respectivement [16].

Le niveau socio-économique et l´âge de la mère étaient associés à l´acceptabilité du dépistage de la drépanocytose chez le nouveau-né. Ainsi, l´acceptabilité était plus élevée chez les mères âgées de plus de 35 ans et chez les mères de bas niveau socio-économique. Concernant le niveau socio-économique, nos résultats rapportent qu´un bas niveau socio-économique était corrélé à l´acceptabilité du dépistage. Au stade actuel de nos connaissances sur la drépanocytose, il n´y a pas une étude réalisée à Kisangani qui peut expliquer cette situation. Cependant, l´exemple de Jatto sur le dépistage néonatal de la surdité, renseigne qu´il y aurait une association significative entre le niveau socio-économique et l´acceptabilité du dépistage néonatal. Dans sa série, les mères de bas niveau socio-économique avaient moins d´information sur le dépistage car ne possédant ni radio, ni télévision ni internet dans les ménages pour s´en informer [23]. Selon notre expérience du terrain, les femmes n´ayant pas des moyens sont parfois faciles à persuader et ont tendance à s´adonner aux mesures préventives par peur de ne pas être capable de faire face aux conséquences des maladies dans le futur.

En rapport avec l´âge des mères, l´analyse multivariée a démontré une corrélation significative entre l´acceptabilité et l´âge de plus de 35 ans. Wang *et al*. [34], ont rapporté que les fréquences réduisent le stress. Ils expliquent que le taux d´acceptabilité est toujours bas au premier contact et augmente avec le contact suivant et progressivement. Nous pensons que l´expérience acquise par une mère avec l´âge et les circonstances, rendent celle-ci moins hostile aux activités préventives et influencent facilement son acceptabilité pour quelque chose qu´elle juge bénéfique pour la santé de son enfant et pour le tissu familial [34]. Il y a certaines limites à cette étude. Premièrement, les mères ayant encore été en période de post-partum immédiat et souvent hospitalisées dans des salles communes, il était difficile d´approfondir leur sentiment lié à la peur au cours de notre passage; deuxièmement, le fait que l´enquêteur devait à la fois sensibiliser et exécuter le test pourrait avoir influencé le résultat au niveau de l'acceptabilité; troisièmement, le fait que notre étude transversale ait été réalisée pendant la phase exponentielle de la pandémie de COVID-19, il nous semble difficile d´extrapoler nos résultats à ceux qui seraient obtenus dans des conditions de vie normale. Ainsi une étude longitudinale sur l'acceptabilité permettra d´aboutir à des résultats reproductibles.

## Conclusion

L´acceptabilité du dépistage néonatal de la drépanocytose par les mères pendant la période de confinement suite à la COVID-19 reste beaucoup trop faible à Kisangani. En plus des problèmes inhérents au dépistage des maladies chroniques, les fausses rumeurs autour de ladite pandémie, les conséquences économiques réelles liées à la pandémie, la difficulté de sensibilisation des mères sur le dépistage de leurs nouveau-nés à la suite du confinement constituent autant d´obstacles à l´implémentation du programme de dépistage. Le gouvernement devrait identifier les canaux de communication efficaces pour promouvoir les initiatives dans le secteur de la santé. Des études multicentriques sont indispensables pour évaluer l´impact réel de la COVID-19 dans la gestion d´autres maladies à prévalence élevée dont la drépanocytose.

### Etat des connaissances sur le sujet

- La drépanocytose représente un problème crucial de Santé Publique dans le monde;
- Sa gestion pendant la pandémie de COVID-19 est difficile;
- Le stress dû à la COVID-19 impacte négativement sur la gestion des endémies existantes dont la drépanocytose.

### Contribution de notre étude à la connaissance

- La pandémie de COVID-19 interfère avec l´acceptabilité, par les mères, du dépistage néonatal de la drépanocytose à Kisangani;
- La difficulté de sensibilisation et la crise économique constituent des obstacles majeurs à l´implémentation des programmes du dépistage de la drépanocytose pendant la pandémie de COVID-19;
- Des stratégies de communication adaptées au contexte de la pandémie de COVID-19 doivent être identifiées afin de promouvoir des initiatives dans le secteur de la santé.

## Remerciements

Les auteurs sont reconnaissants à l´égard des mères des enfants, des responsables de différentes structures et des maternités pour leur consentement à participer à la réalisation de cette étude. Ils sont également reconnaissants à l´égard de l´Académie de Recherche et de l´Enseignement Supérieur de la Coopération Belge au Développement pour l´appui au projet PRD-DREPAKIS et au projet Synergie Save Drepachild.
